# An Extended N-Player Network Game and Simulation of Four Investment Strategies on a Complex Innovation Network

**DOI:** 10.1371/journal.pone.0145407

**Published:** 2016-01-08

**Authors:** Wen Zhou, Nikita Koptyug, Shutao Ye, Yifan Jia, Xiaolong Lu

**Affiliations:** 1 School of Computer Engineering and Science, Shanghai University, Shanghai, China; 2 Kellogg School of Management, Northwestern Institute on Complex Systems (NICO), Northwestern University, Evanston, Illinois, United States of America; 3 Research Institute of Industrial Economics (IFN), Stockholm, Sweden; Nankai University, CHINA

## Abstract

As computer science and complex network theory develop, non-cooperative games and their formation and application on complex networks have been important research topics. In the inter-firm innovation network, it is a typical game behavior for firms to invest in their alliance partners. Accounting for the possibility that firms can be resource constrained, this paper analyzes a coordination game using the Nash bargaining solution as allocation rules between firms in an inter-firm innovation network. We build an extended inter-firm n-player game based on nonidealized conditions, describe four investment strategies and simulate the strategies on an inter-firm innovation network in order to compare their performance. By analyzing the results of our experiments, we find that our proposed greedy strategy is the best-performing in most situations. We hope this study provides a theoretical insight into how firms make investment decisions.

## Introduction

Game theory plays a significant role in economics with many economic phenomena having been modelled as games [1–4]. There are many different types of games and these can differ in two main dimensions; games can differ in the number of participants–for instance two-player or n-player games [5,6]–and whether the participating players can credibly commit to a set of actions, more generally known as cooperative and non-cooperative games [7]. One core problem of any game is to compute the Nash equilibrium—one of the most fundamental and central concepts in game theory which provides a solid foundation for generalizing game theory. The Nash equilibrium concept is named after John Nash who provided the first existence proof in finite games by using Brouwer’s fixed point theorem.

After the establishment of the Nash equilibrium concept, researchers have provided many methods for calculating the Nash equilibria in games; a non-linear optimization model was proposed to compute Nash equilibria in finite games, and the algorithm based on the quasi-Newton technique was coded in MATLAB by using sequential quadratic programming [8]. A method for computing the Nash equilibrium within a class of generalized Nash equilibrium problems with shared constraints through fixed point formulation has also been developed [9]. Finally, another method to solve generalized Nash equilibrium problems uses parametrized variational inequality approaches [10].

In this paper, we study the Nash equilibrium on a complex innovation network. An innovation network is a social network with specific meanings and objectives—a network formed among firms for the purpose of innovation and knowledge sharing [11]. The firms in an innovation network are connected by way of alliances, with the alliance being able to enhance their own accumulation of knowledge and skill level [12]. Therefore, innovation networks can also be referred to as alliance networks. With the development of complex network research, scholars have been able to find complexity in innovation networks; empirical research by Verspagen and Duysters confirmed that innovation networks based on a strategic alliance have the so-called small-world property [13]. There are other measures characterizing the structural properties of networks including entropy and distance measures [14–16]. In this paper, we refer to an innovation network that satisfies the small-world property and is scale free as a complex innovation network.

Innovation networks are able to achieve a solution in which resources are allocated optimally [17]. Social network structure is the key of information dissemination and innovation [18]. A lot of researchers have concentrated on the relationship between innovation network structure and the level of innovation within the network [19–21]. In order to carry out empirical research in this area, researchers compared the alliance network structure in different industries and as a consequence been able to make recommendations about how to use different databases, how to combine first-hand and secondary data as well as exploring data sampling issues [22,23]. Some specific network structures which are more regular were studied by Lovejoy and Sinha. They point out that a complete graph and network structure with a core can promote the early formation of ideas for innovation [24]. Through research on innovation networks, it may be possible to understand the relationship between network attributes and the degree of innovation, and can provide a theoretical basis for improved strategic decisions. This paper has constructed and simulated a game in order to study innovation networks from the perspective of firms.

In game theory, a strategy refers to a specific set of actions taken by a player, with different strategies potentially leading to different outcomes or payoffs. Strategies have been studied in different fields such as economics, politics and warfare. One widely studied strategy is known as the Tit For Tat (TFT) strategy [25]. Especially in repeated games, the TFT is an efficient strategy that can be used to promote cooperation. In the TFT strategy, a player always chooses cooperation during the first round of a game, and then imitates its opponent’s strategy in subsequent rounds. The TFT strategy which gives a solution to 2-player prisoner's dilemma game is based on the unrealistic assumption that all players observe the actions of all other players. To overcome this shortage, Nakai and Muto proposed the us-Tit For Tat (us-TFT) strategy that requires a player to regard another player who cooperated with himself or his partners as a friend and showed that this strategy lead to an emergence of a mutually cooperative society [26]. This is more realistic as players playing us-TFT need only observe what has occurred to himself and his allies rather than the entire set of players. If the players use responsive strategies such as TFT, Roberts and Sherratt find that it is difficult to solve the fundamental question of how altruistic one should be when they simulate the prisoner's dilemma game. They propose the 'raise-the-stakes' (RTS) strategy based on a variable investment [27]. This strategy proposes that players offer a small amount in the first round and then, if matched, the firm raises its investment, something that no strategy in the discrete model can do. The analysis has also been extended to study cooperation in different kinds of social dilemmas from a dynamic, rather than static, perspective [28–30].

It is possible to express network games more compactly than normal games. The scale index of a network game is restricted by its adjacent node, if compared to a normal game. Research related to networks is based on graph theory, a branch of discrete mathematics, now largely used to understand the formation of all kinds of networks and the effect network structure has on member behavior. Network games are also widely applied within the field of economics. Jackson uses a game to study how economic networks are formed [31]. When a network game is applied to describe different environments, the features of the network structure and the position of network members have different effects on members’ behavior and payoff [32]. A complex network game can be used to study how local externalities shape the strategic behavior of players when the underlying network is volatile and complex [33]. Some scholars have researched how players should choose payoff-maximizing strategies within the setting of a complex network [34–36]. In a network game, every agent is regarded as a player in a non-cooperative game. Each player rationally chooses the strategy to maximize the object function (pursue maximum payoff). In doing so, all players can achieve a Nash equilibrium whereby the network reaches a steady state and no player can benefit by deviating from his optimal strategy.

In this paper, we present an extended n-player game under non-idealized conditions, namely that players are resource-constrained. In real social situations, firms do not tend to invest in their allies according to the theoretical unconstrained Nash equilibrium solution because of financial, human or other resource constraints. This means that firms need to change their investment strategies based on the resource constraints they face. This paper provides four extreme strategies for allocating resources among alliance partners including an average strategy, a proportional strategy, a greedy strategy and a random strategy. The advantages and disadvantages of the four strategies are compared in order to determine which strategy should be used in the firms’ investment decision process. For each strategy, we establish and simulate an experimental model and draw the conclusion that, most often, a greedy strategy offers the best performance.

## Methods

In this section we extend the classic business partnership game to allow for a variable project return ratio and describe and analyze its Nash equilibrium. Thereafter we extend the two-player case to an n-player network game in which alliance firms must choose how to allocate their resources among partner firms under both idealized and nonidealized conditions.

### An extended two-player game and its Nash equilibrium

Allying with other firms can promote innovation for a firm, but some investment is required in order to form strategic alliances. The level of investment that is required does not only depend on the firm itself but is also influenced by how much the partner firm is planning to invest—a typical game behavior. In this section, we extend the classic business partnership game (see S1 File for a description of the game and equilibrium strategies) to allow for differences in the return ratios of their investments.

Assume two firms, 1 and 2, which are cooperating on a mutual project. Assume that for this project to be successful both firms need to invest, but having done so they divide any profits equally–a kind of win-win relationship. In the classic partnership game, the return ratio of the investment is set to 4. Van Zandt sets this parameter to 16 in his partnership game [37]. We set this parameter as a variable *γ* giving income as Eq (1)

$$I=\gamma \;(S_{1}+S_{2}+bS_{1}S_{2})$$ (1)

*S*_1_ and *S*_2_ are the both firms’ investment respectively. Let parameter *b*, known as the complementary coefficient, be non-random and common knowledge among the firms. Further, let the complementary coefficient be restricted to values between 0 and $\frac{1}{4}$, that is let $b\in \left[0,\frac{1}{4}\right]$. From Eq (1), we see that the total payoff received by the two firms depends on both firms’ strategies *S*_1_ and *S*_2_ and the synergy, or cooperative effect given by *bS*_1_*S*_2_, that is generated by the two firms working together. In reality, firms have different competitive advantages and are skilled at different projects. This is why mutual investment and cooperation may lead alliances to generate additional income as compared to the income they could have generated individually. Assuming income *I* is split equally between the two firms and that investment cost is quadratic in the level of investment, the payoff expressions *P*_1_ and *P*_2_ corresponding to firm 1 and firm 2 are given by the system of equations in (2).

$$\left\{ \begin{array}{l} P_{1}=\frac{1}{2}*\gamma \;(S_{1}+S_{2}+bS_{1}S_{2})-S_{1}^{2}\\ P_{2}=\frac{1}{2}*\gamma \;(S_{1}+S_{2}+bS_{1}S_{2})-S_{2}^{2} \end{array}\right.$$ (2)

Given their payoff functions, firms need to choose their optimal strategies $\left(\hat{S_{1}},\hat{S_{2}}\right)$ in such a way that any firm’s strategy is a best response to the other firm’s strategy. The first firm needs to find the best response strategy $\hat{S_{1}}$ based on the strategy *S*_2_ that the second firm chooses. Similarly, the second firm needs to find the best response strategy $\hat{S_{2}}$ based on the strategy *S*_1_ of the first firm.

In order to find the best responses for both firms, let us first compute the first-order partial derivative of *P*_*i*_ with respect to *S*_*i*_, giving

$$P_{i}^{\prime }=\frac{1}{2}*\gamma \;(1+bS_{j})-2S_{i}$$ (3)

Setting the derivatives in (3) to zero, $P_{i}^{\prime }=0$, we find that each firm’s best-response function is given by $S_{i}=\frac{1}{4}*\gamma *(1+bS_{j})$. Let *BR*_*i*_(*S*_*j*_) denote the best response that *i* takes when *j* adopts strategy *S*_*j*_, then the best response functions of 1 and 2 are given by (4).

$$\left\{ \begin{array}{l} {BR}_{1}(S_{2})=\hat{S_{1}}=\frac{1}{4}*\gamma *(1+bS_{2})\\ {BR}_{2}(S_{1})=\hat{S_{2}}=\frac{1}{4}*\gamma *(1+bS_{1}) \end{array}\right.$$ (4)

As can be seen from (4), if $S_{i}<\frac{1}{4}*\gamma *(1+bS_{j})$, firm *i* does not have the resources to achieve the maximum, theoretical payoff. On the other hand, if *S*_*i*_ > 1 + *bS*_*j*_, firm *j* may not be able not increase its level of investment *S*_*j*_ and the resources that firm *i* invested are wasted. This means that only if $S_{i}=\frac{1}{4}*\gamma *(1+bS_{j})$ can firm *i* achieve the maximal payoff. Let $S_{i}^{*}$ denote the Nash equilibrium solution, then we can easily verify the Nash equilibrium given by (5).

$$S_{1}^{*}=S_{2}^{*}=\frac{\gamma }{4-\gamma b}$$ (5)

In this state, no firm can benefit by deviated from its strategy given the strategy of the other firm and hence the current set of strategies $\left(S_{1}^{*},S_{2}^{*}\right)$ constitute a Nash equilibrium.

From this result, we see that when the complementary coefficient *b* diminishes, meaning that the returns to collaboration diminish, the payoffs will decline for both sides. The firm that invests more incurs a higher marginal cost but only receives half of the marginal return.

Increasing the return ratio *γ* has a similar effect to increasing the complementarity coefficient as increasing the return to any of the firms participating in a joint project increases the level that the firms with to invest in the partnership.

**Fig 1:**
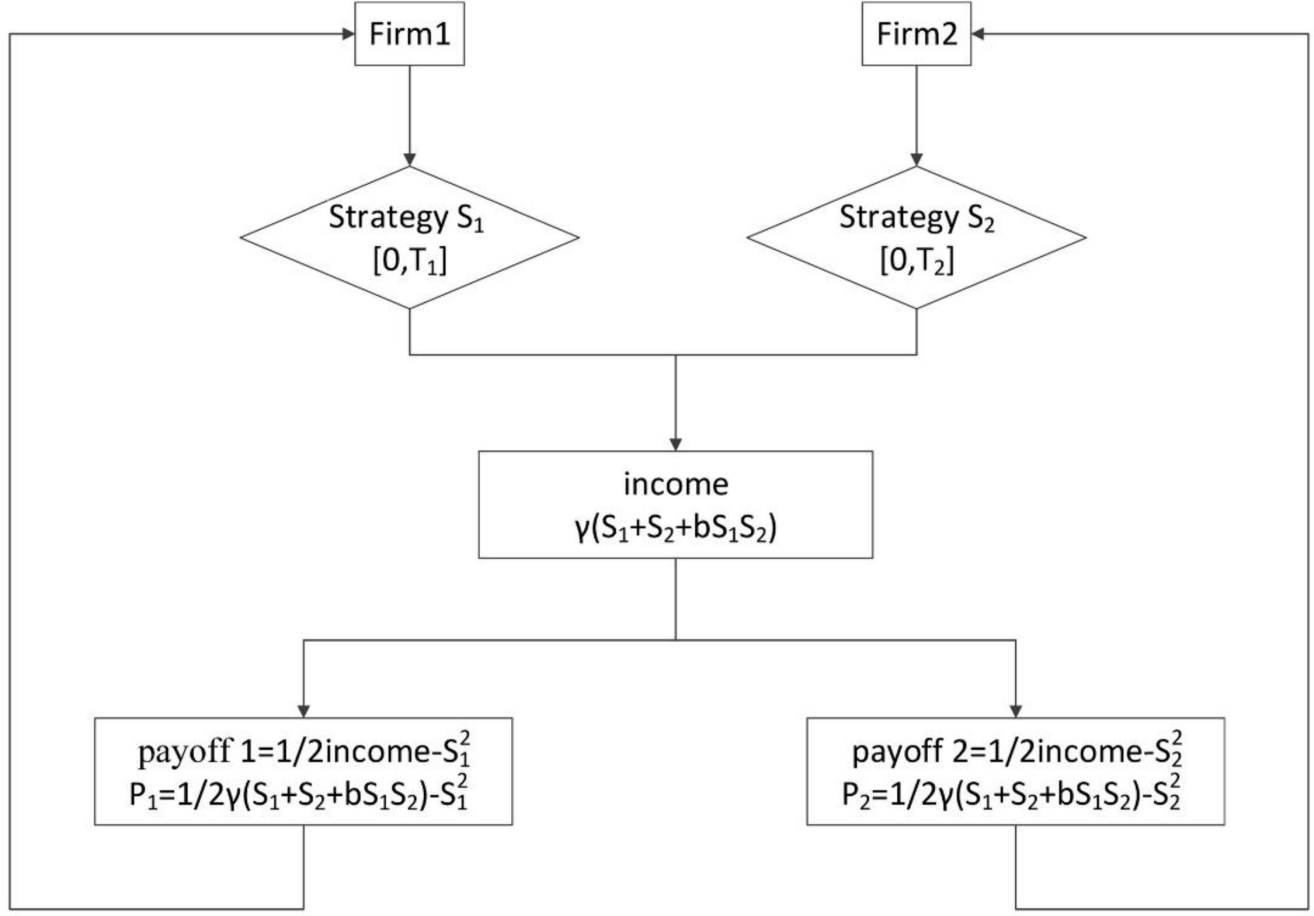
Flow chart of investment income of two firms.

The flow chart of an extended two-player game is shown in Fig 1.

### An extended n-player network game under idealized conditions

The n-player game has a certain number of players and we will use a network to represent the relationships between these players. Let *V* = {1,2, …, *n*} be the set of nodes and let *E* = {*e*_*ij*_}(*i*,*j* ∈ *V*) be the set of edges. A network can be thought of as an undirected graph and represented as *G* = {*V*,*E*}. The set *V* is also a set of players. Let *e*_*ij*_ = {0,1}. Then *e*_*ij*_ = 1 represents an edge between the node *i* and *j*. *V*(*i*) = {*j*|*e*_*ij*_ = 1} shows all the neighbors connected with *i*, and the number of the direct neighbors is called the degree of *i*, *d*_*i*_ = |*V*(*i*)|. In this model, each node represents a firm. Firm *i* plays strategy *X*_*i*_, where *x*_*i*_ denotes the realization of *X*_*i*_ and is a non-negative real number. The payoff of firm *i* can be represented as a vector *m*_*i*_(*x*_*i*_,*x*_*V*(*i*)_) where *x*_*V*(*i*)_ is the vector of actions taken by the partners of firm *i*. As before, the payoff of firm *i* depends on the actions of its partners and on its own actions. Letting *d*_*i*_ = *k*, the payoff vector of firm *i* and its action vector *X*_*i*_ is given by Eq (6).

$$m_{i}(x_{i},x_{1},\ldots ,x_{k})=f(x_{i}+\lambda \sum_{j=1}^{k}x_{j})-c(x_{i})$$ (6)

In Eq (6), let *f*(∙) be a non-decreasing function and *c*(∙) the cost function associated with the investment of firm *i*. The parameter λ is set to 1. Then the network game is fully characterized by (*G*,*X*,*m*_*i*_).

Assume that under idealized conditions, each firm has enough resources and can fully meet the needs of its partners. This network can reach a Nash equilibrium; Assume that firm *i* and its partner *j* ∈ *V*(*i*) can reach Nash equilibrium with the heterogeneous return ratios *γ*_*ij*_ and complementary coefficients *b*_*ij*_. As before, $S_{ij}^{*}=\frac{\gamma_{ij}}{4-\gamma_{ij}b_{ij}}$ is the best investment strategy within each partnership. As for firm *F*_*i*_, $S_{ij}^{*}$ denotes the Nash equilibrium solution between firm *F*_*i*_ and *F*_*j*_. Therefore, the total investment *R*_*i*_ of firm *i* is given by Eq (7).

$$R_{i}=\sum_{j=1,j\neq i}^{degree(F_{i})}S_{ij}^{*}=\sum_{j=1,j\neq i}^{degree(F_{i})}\frac{\gamma_{ij}}{4-\gamma_{ij}b_{ij}}$$ (7)

Also, the payoff expression *P*_*i*_ is given by (8).

$$P_{i}=\sum_{j=1,j\neq i}^{degree(R_{i})}P_{ij}=\sum_{j=1,j\neq i}^{degree(R_{i})}[\frac{1}{2}*\gamma_{ij}\;(S_{ij}+S_{ji}+b_{ij}S_{ij}S_{ji})-S_{ij}^{2}]$$ (8)

**Fig 2:**
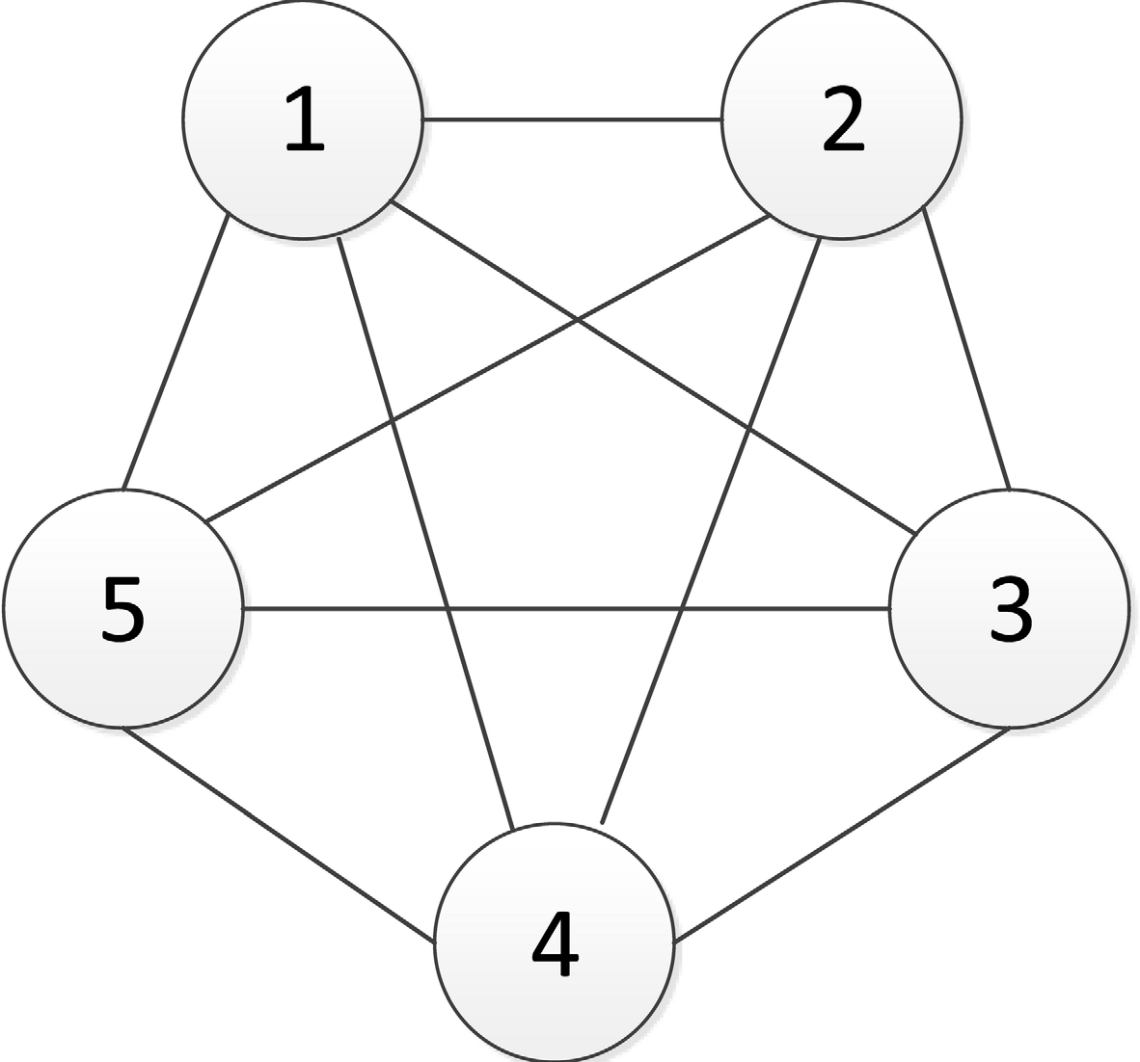
The network diagram of the first five firms of *G*_*2001*_.

As shown in Fig 2, we take the *G*_*2001*_ innovation network diagram for the automobile industry during 2001–2003 as an example and firms No.1 to No.5 happen to constitute a complete graph of five elements.

Let us look at firm No.1, the degree *d*_1_ of which is 4. Assuming that the return ratios of firm No.1 are *γ*_12_, …, *γ*_15_ and the complement coefficients are *b*_12_, …, *b*_15_ we can calculate the required initial investments of firm No.1 $R_{1}=S_{12}^{*}+S_{13}^{*}+S_{14}^{*}+S_{15}^{*}$ and the payoff *P*_1_ = *P*_12_ + *P*_13_ + *P*_14_ + *P*_15_ given that the level of initial investments are the Nash equilibrium solution $S_{12}^{*},\ldots ,{\;S}_{15}^{*}$.

### An extended n-player network game under non-idealized conditions

#### Non-idealized conditions: resource constraints

Under idealized conditions, the total investment *R*_*i*_ a firm would like to invest is merely the theoretical value given by the Nash equilibrium solution given in (7). In reality, some firms’ resource reserves cannot fully meet the theoretical *R*_*i*_ for various reasons. When firm *i*’s resources *T*_*i*_ cannot meet partners’ resources requirements in a Nash equilibrium state (*T*_*i*_ < *R*_*i*_), a firm’s investment to its partners according to the Nash equilibrium will be greater than its own resources reserves. This kind of situation is called a resource shortage or constraint. Let the extent of the resource shortage Δ_*i*_ be defined as in (9) and the average resource shortage per degree *θ*_*i*_ as in (10).

$$\Delta_{i}=|T_{i}-R_{i}|$$ (9)

$$\theta_{i}=\Delta_{i}/d_{i}$$ (10)

Under a resource shortage, firms will employ different strategies to cope with the shortage of resources and control their total level of investment. During the process of adjustment of these firms, their partners will also adjust their strategies to maintain the best countermeasures to the new Nash equilibrium. Assume that two allied firms *i* and *j* have the initial strategies given by $S_{ij}^{*}=S_{ji}^{*}$ and that each firm’s level of resources *T* is common knowledge. If firm *j* changes its strategy to a new investment strategy $S_{ji}^{\prime }$ due to a resource shortage then according to Eq (4) firm *i*’s best response given the new strategy is ${BR}_{i}(S_{ji}^{\prime })$. So for firm *i*, the required total investment $R_{i}^{\prime }$ will be adjusted accordingly, as shown by Eq (11).

$$R_{i}^{\prime }=R_{i}-(S_{ij}^{*}-{BR}_{i}(S_{ji}^{\prime }))$$ (11)

In this paper, we propose four investment strategies when firms face a shortage of resources and through simulation of a real alliance network we assess the outcomes of the four strategies by analyzing total investment, total payoff, the average return ratio, the degree of the average return ratio and assess their overall advantages and disadvantages.

#### Strategy 1: the average strategy

The idea of the average strategy is that when a firm faces a shortage of resources it will assign all of its resources equally to its partners, namely the inputs to each partner are $S_{ij}^{\prime }=\frac{T_{i}}{d_{i}}$. Additionally, each partner *j* will optimally adjust its own investment. The average strategy algorithm is given below:

Step 1: Initialize the network based on the Nash equilibrium of every partnership;
Step 2: Mark all the firms with a shortage of resources as *i*, find out the extent of the resource shortage Δ_*i*_ and the average resource shortage per degree *θ*_*i*_;
Step 3: Sort the average resource shortage per degree *θ*_*i*_ in descending order;
Step 4: Take the largest *θ*_*i*_ firm *i*, invest its resources equally to its partners according to $S_{ij}^{\prime }$. At the same time adjust the investment of its partners *j* to ${BR}_{i}(S_{ji}^{\prime })$ and modify the total resource investment to $R_{j}^{\prime }$. Mark *i* as a treated firm.
Step 5: For the remaining firms complete Steps 2–4, until there is no shortage of resources in the network.

The time complexity of the average strategy is *O*(*n*^2^). Under the assumption that each firm’s available resources are common knowledge the allies of a resource constrained firm will be able to change their own strategies to a firm playing the average strategy. For the firms which face a resources shortage in the first allocation, after adjusting, the total investment may turn out to be within acceptable limits and the firm’s resource budget is sufficient again. From a practical point of view, the firm whose shortage of resources is more serious has to take the lead in the adjustment process.

#### Strategy 2: the proportional strategy

The proportional strategy is when a firm with a shortage of resources reduces the investment to its partners according to the fixed proportion $q=\frac{T_{i}}{R_{i}}$, and the reduced investment becomes $S_{ij}^{\prime }=S_{ij}*q$ where *S*_*ij*_ is the initial, unconstrained Nash equilibrium. The algorithm for calculating the outcome of the proportional strategy is given below:

Steps 1–3: As in Strategy 1.
Steps 4: Take the largest *θ*_*i*_ firm *i* and with the same proportion *q* reduce its investment to all partners to $S_{ij}^{\prime }$. At the same time adjust the investment of its partners *j* to ${BR}_{i}(S_{ji}^{\prime })$ and modify the total resource investment to $R_{j}^{\prime }$. Mark *i* as a treated firm.
Steps 5: As in Strategy 1.

The time complexity of the proportional strategy is *O*(*n*^2^).

#### Strategy 3: the greedy strategy

The greedy strategy is when a firm’s investment strategy is equal to the needs prescribed by the unconstrained Nash equilibrium, each time meeting the needs of the partner whose return ratio is highest, until the investing firm finally runs out of resources.

Assume that under idealized conditions firm *i*’s investment into firm *j* is $S_{ij}^{*}$ and that the current investment is *S*_*ij*_. Further, let firm *j*’s investment into firm *i* be *S*_*ji*_. The current total investment is *R*_*j*_. Depending on the firms’ situation, specific allocation strategies are shown in Table 1.

**Table 1:** Greedy strategy investment-adjustment table.

Situation	Firm *i*	Firm *j*	Processing Method
1	*S*_*ij*_ = $S_{ij}^{*}$	*S*_*ji*_ = $S_{ij}^{*}$	Do not adjust
2	0<*S*_*ij*_<$S_{ij}^{*}$	*S*_*ji*_ = $S_{ij}^{*}$	*S*_*ij*_ does not adjust; *R*_*j*_ adjusts to $R_{j}^{\prime }$ (Eq 11, the same below)
3	*S*_*ij*_ = $S_{ij}^{*}$	0<*S*_*ji*_<$S_{ij}^{*}$	*S*_*ij*_ adjusts to *BR*_*i*_(*S*_*ji*_); *R*_*j*_ adjusts to $R_{j}^{\prime };\;\Delta S_{ij}$ allocate in turn as *List*(*i*)
4	0<*S*_*ij*_<$S_{ij}^{*}$ and *S*_*ij*_ > *BR*_*i*_(*S*_*ji*_)	0<*S*_*ji*_<$S_{ij}^{*}$	*S*_*ij*_ adjusts to *BR*_*i*_(*S*_*ji*_); *R*_*j*_ adjusts to $R_{j}^{\prime };\;\Delta S_{ij}$ allocate in turn as *List*(*i*)
5	0<*S*_*ij*_<$S_{ij}^{*}$ and *S*_*ij*_ < *BR*_*i*_(*S*_*ji*_)	0<*S*_*ji*_<$S_{ij}^{*}$	*S*_*ij*_ does not adjust; *R*_*j*_ adjusts to $R_{j}^{\prime };$

The algorithm for calculating the greedy strategy is given below;

Step 1: For each firm in the network, sort its partners in descending order according to the return ratio *γ* (if equal then sort in descending order according to complementary coefficient *b*) in order to obtain the sequence of investment *List*(*i*). Every firm invests in turn according to the sequence *List*(*i*) on the basis of the Nash equilibrium. Proceed until completed or resources run out.
Steps 2–3: As in Strategy 1.
Step 4: Take the largest *θ*_*i*_ firm *i*, invest in turn according to the sequence in *List*(*i*). Mark *i* as a treated firm.
Step 5: For the remaining firms, complete Steps 2–3, until there is no shortage of resources in the network.
Step 6: For any remaining untreated firm *i* (with sufficient reserves) fix the level of investment. If its partner *j* is the treated firm, and firm *i*’s investment into firm *j* is $S_{ji}^{\prime }$ right now, revise firm *i*’s investment to the best response ${BR}_{i}(S_{ji}^{\prime })$.

The time complexity of the greedy strategy is *O*(*n*^3^).

#### Strategy 4: the random strategy

In contrast to the three allocation strategies above, the random strategy selects random partners to invest resources in all of its relationship and the sum of the investments is equal to the firm’s resource reserves value. The algorithm for calculating the random strategy is given below;

Steps 1–3: As in Strategy 1.
Step 4: For all firms *i* facing a resource shortage, invest $S_{ij}^{\prime }$ randomly in the relationship with firm *j*. The total value of the investments is the firm’s resource reserves. At the same time adjust the investment of its partners *j* to ${BR}_{j}(S_{ij}^{\prime })$, and modify the total resource investment to $R_{j}^{\prime }$. Mark *i* as a treated firm;
Step 5: For the reaming firms complete Steps 2–4, until there is no shortage of resources in the network.

The time complexity of the random strategy is *O*(*n*^2^).

These four strategies can all be seen as extreme adjustment strategies. In the real world, because there are many factors to consider, firms cannot completely adjust according to these strategies. However, we believe that these strategies provide a reference point for the firm’s adjustment strategy and that firms can make investment strategies based on these.

## Results and Discussion

### Data and experiment

The data used in the experiment is for the automotive industry innovation network of Chinese automobile manufacturing firms *G*_*2001*_ and is taken from the Thomson Reuters SDC Platinum database for the 2001–2003 period. The data is manually compared with Chinese news to add any missing data and correct any issues in the existing data [38]. The *G*_*2001*_ network is built from this data in the 3 year interval using the fast innovation network building method described in [39]. The data and a corresponding description can be found in S2 File and S3 File respectively.

The network consists of a total of 54 node firms and 66 cooperative relationships. The parameters needed for conducting the experiment are the return ratio *γ*, the complementary coefficient *b*, each firm’s resource reserves *T* and each firm’s total investment *R*. In the *G*_*2001*_ data, the return ratio, the coefficient of complementary or firm resource budget information are all unavailable, so we set these parameters through randomization.

The return ratio *γ* and the complementary coefficient *b* depict each edge of the network and the parameters of each edge should be different. The return ratio and complementary coefficient data between the alliance is not available and we cannot use other indicators in their place. For the resource budget of the firm, we need to consider the firm’s own resources and the amount the firm is willing to invest into the network. In this section, we use the firm’s degree in the alliance to adjust the resource budget *T*. Each firm’s total investment *R* in the innovation network can be calculated through each relationship’s *γ* and *b* parameters.

**Fig 3:**
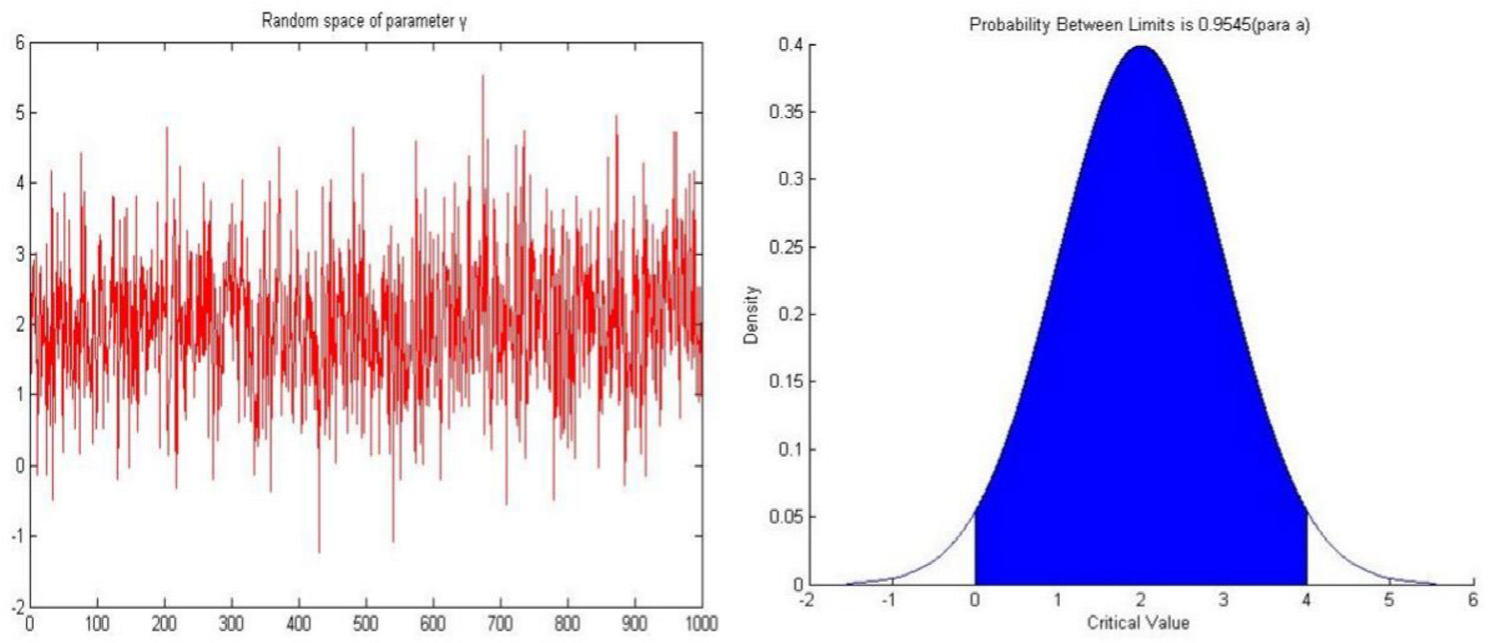
The parameter space of parameter *γ*.

In previous studies, the value of the return ratio *γ* is typically 2 but because the value of parameter *γ* on every edge in the innovation network should be different, in this experiment we assign *γ* randomly. The random space of parameter *γ* includes 1000 random values, these random values are drawn from a normal distribution (*μ* = 2, *σ* = 1), the values are mainly concentrated between 0 and 4 (*p* = 0.9545) and we replace the randomly generated negative values with 0. Fig 3 depicts the parameter space of *γ*.

**Fig 4:**
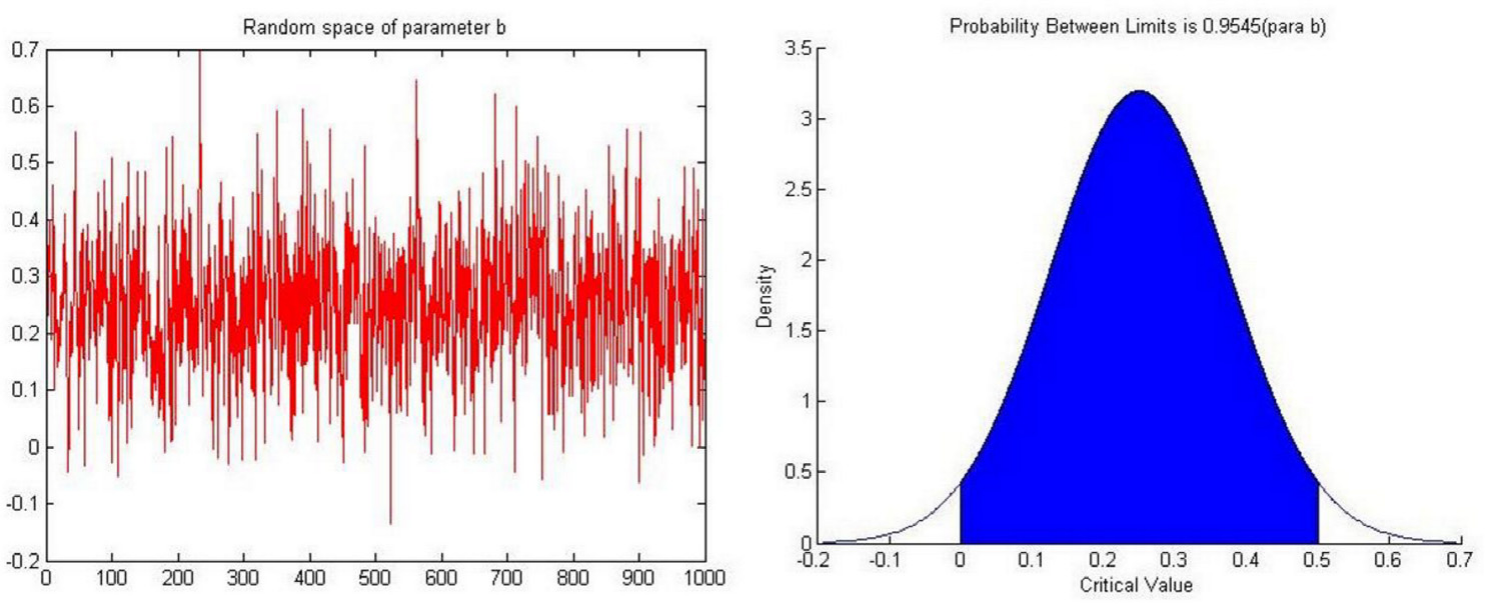
The parameter space of parameter *b*.

In previous research, the value of the complementary coefficient *b* is typically set to $\frac{1}{4}$ but in this experiment parameter *b* comes from a random parameter space containing 1000 values. These random values are drawn from a normal distribution ($\mu =\frac{1}{4},\sigma =\frac{1}{8}$), the values are mainly concentrated between 0 to 1/2 (*p* = 0.9545) and again we set negative values to 0. Fig 4 depicts the parameter space of *b*.

**Fig 5:**
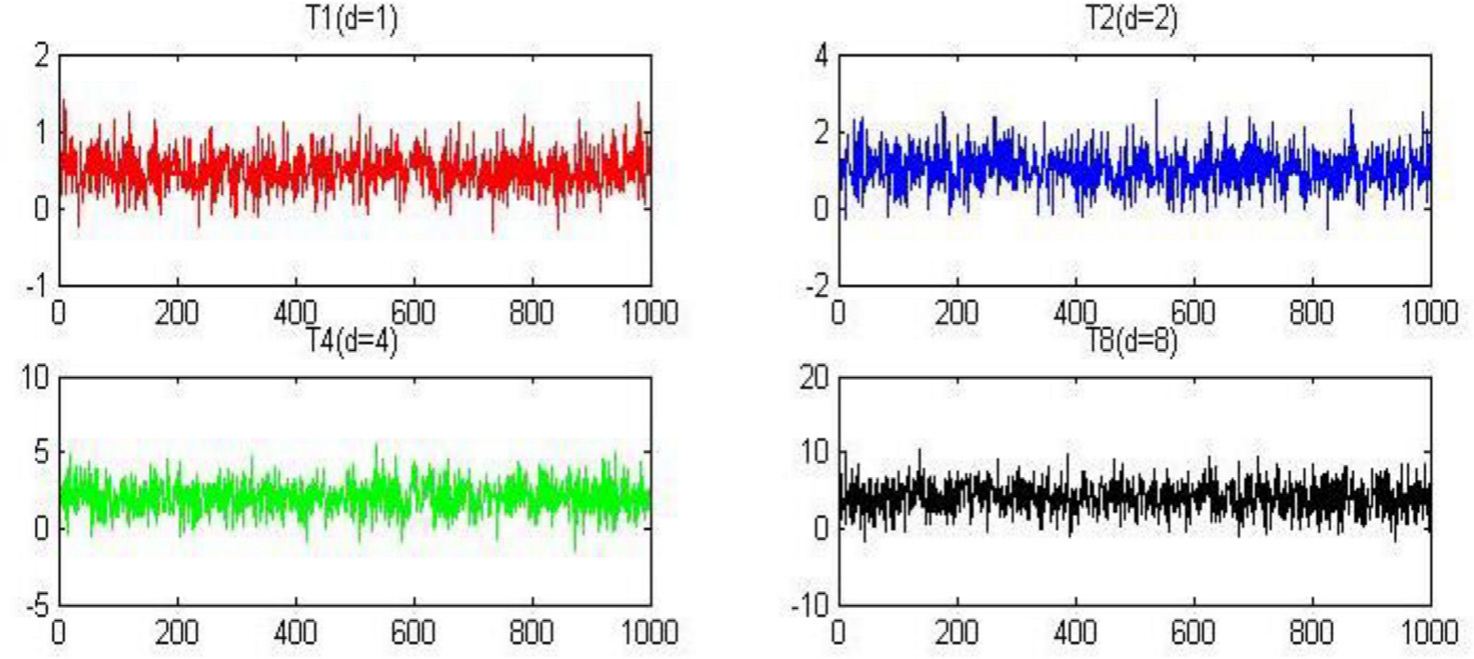
The parameter space of *T* under different node degrees.

The resource budget of each firm is independent of the parameters *γ* and *b*. The resource budget is the value of a firm’s capital input to the alliance and the firm can set it according to its own willingness. However, the capital firm’s typically allocate is both uncertain and unpredictable. One simple way is to relate a firm’s capital allocation towards alliance investment as a function of the number of firms within each firm’s current alliance (that is, the degree of the firm in the innovation network). In this experiment, we randomly generate a resource budget for each node and adjust the random value through the degree of the node. We draw random resource budgets from a normal distribution and assuming that the degree of the firm node is *d*, we let the resource budget of the firm node be concentrated on the interval [0,*d*]. If we set ($\mu =\frac{d}{2},\sigma =\frac{d}{4}$) then the probability that the value of resources budget randomly generated within [0,*d*] is 0.9545. Again, we set negative values to 0. Fig 5 shows the parameter space of *T* under 4 different degree values.

The investment demand of the network can be calculated by the Nash equilibrium of each relationship. After generating the return ratio *γ*, complementary coefficient *b*, firm resource budget *T* and a firm’s total investment demand *R*, we can analyze and discuss the advantages and disadvantages of these four resource allocation strategies.

In this experiment, we used the controlling variable method. We made single variable changes to the return ratio *γ*, complementary coefficient *b*, firm resource budget *T* respectively, and analyze the possible impact of these single factor changes on the results of the experiment. In order to improve the credibility of the experiment and improve accuracy, this experiment is replicated multiple times to make the results more representative (each individual group experiment is replicated 50 times). This experiment prepared 3 groups with a total of 150 times the initial data.

### Experimental results

The change in the experimental parameters—return ratio *γ*, complementary coefficient *b* and firm resource budget *T*, can substantially influence each firm’s investment and income. When we modify the return ratio *γ* or complementary coefficient *b*, each firm’s total investment expenditure *R* will change according to the formula $\frac{\gamma }{4-\gamma b}$, and subsequently, the income can be calculated according to the allocation strategy. When we instead modify the firm resource budget *T* the degree to which a firm faces a resource shortage will change. We can obtain the corresponding income levels according to the allocation strategy.

**Fig 6:**
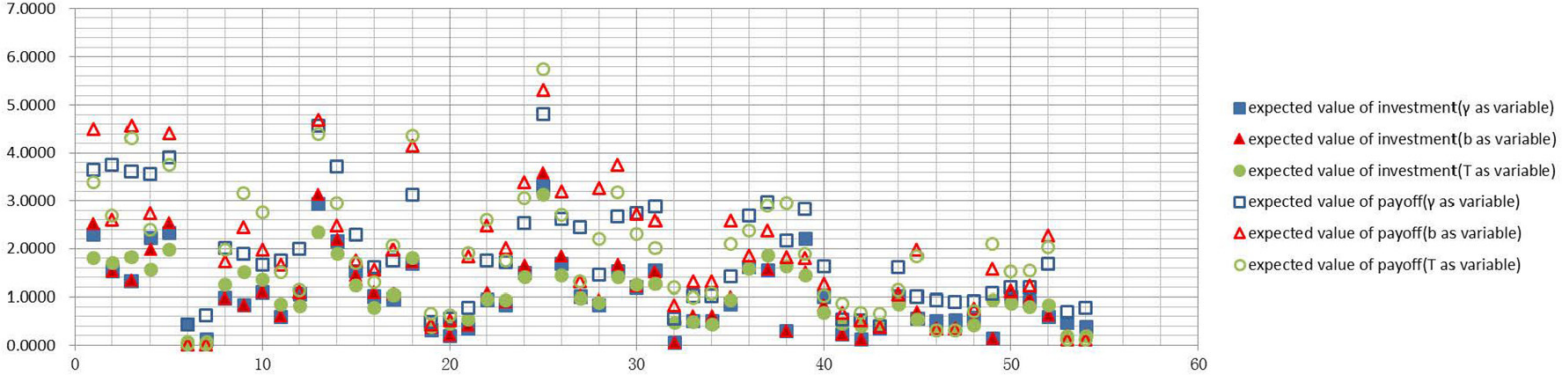
Investment and income for the average strategy.

**Fig 7:**
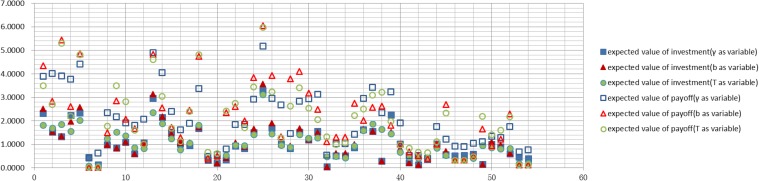
Investment and income for the proportional strategy.

**Fig 8:**
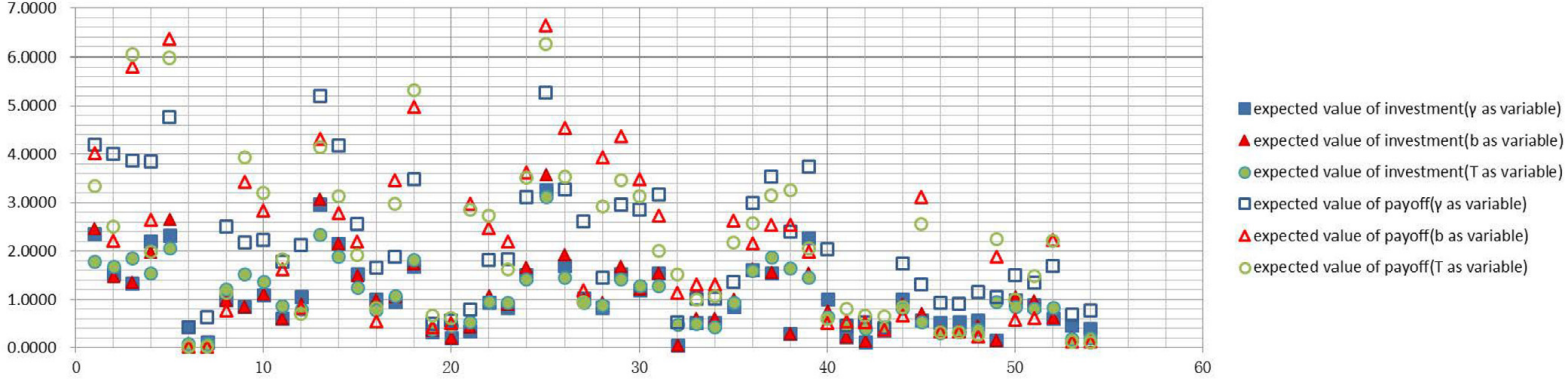
Investment and income for the greedy strategy.

**Fig 9:**
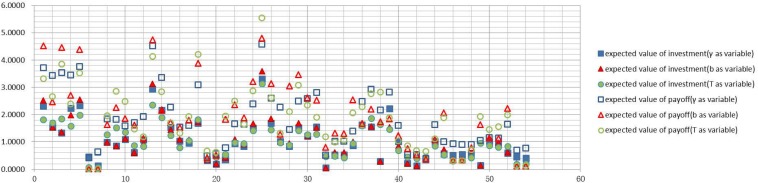
Investment and income for the random strategy.

Figs 6–9 graph the experimental results and show the expected value of income and expenses for the four allocation strategies proposed earlier. In these figures, the solid icons show the firm’s mathematical expectation of the level of investment whereas hollow icons represent the mathematical expectation of the income.

1. Experimental results of every firm node for the average strategy
2. Experimental results of every firm node for the proportional strategy
3. Experimental results of every firm node for the greedy strategy
4. Experimental results of every firm node for the random strategy

Innovation networks are composed of alliance firms and their allied relationships. On the one hand, the members of an organized network always want to pursue the best interests of the alliance; On the other hand, the firm also wants to prioritize the status and interests of the firm itself. Therefore, we can compare and analyze the experimental results from the point of view of the whole network and the network node, the firm.

#### Contrast and analysis of network level

**Fig 10:**
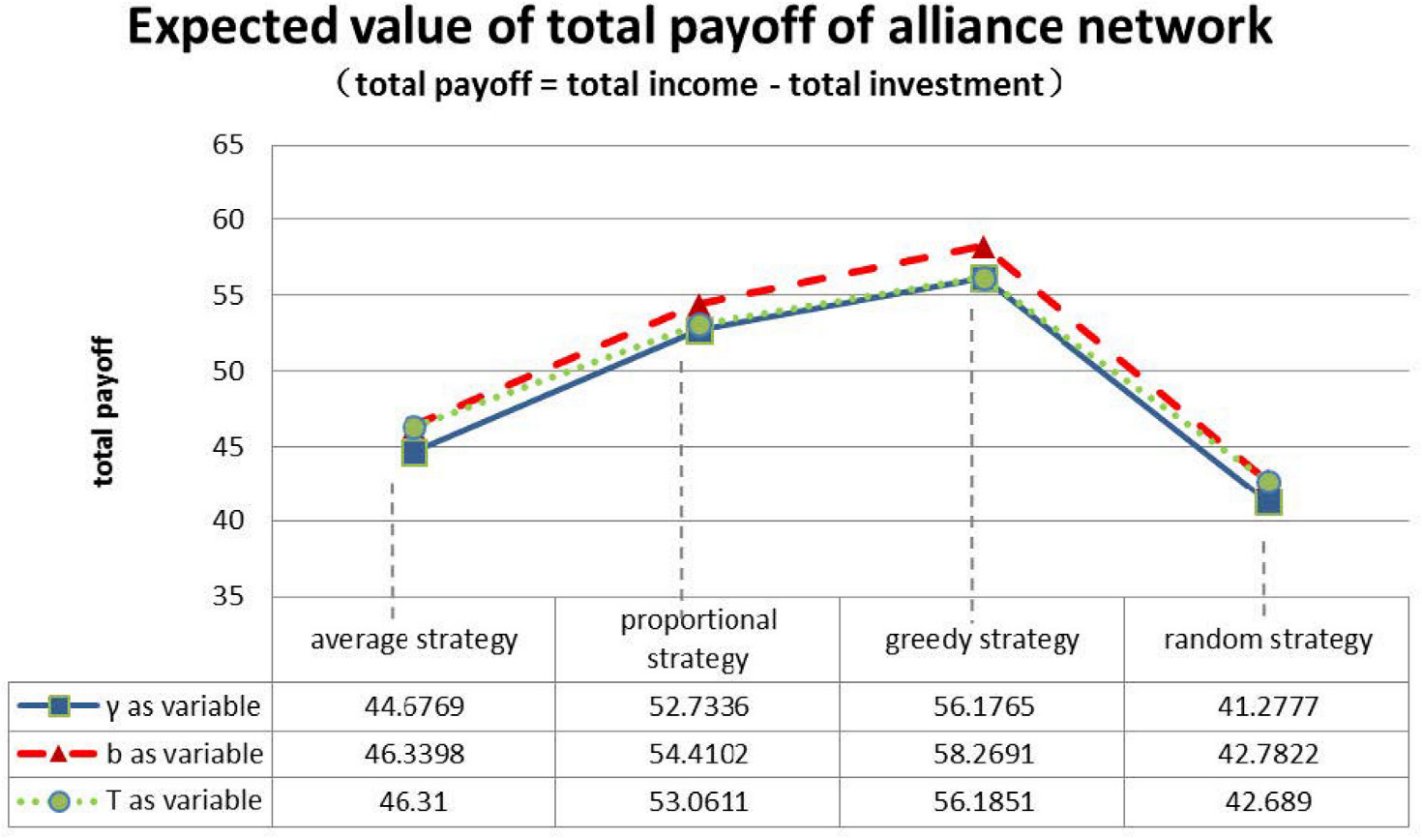
Profit expectations under four allocation strategies.

First of all, from the perspective of the whole network, we compare the total payoff expectations of the three groups of parameters for the initial game for each of the four strategies. The results are shown in Fig 10.

As we can see from Fig 10, the return ratio *γ*, complementary coefficient *b* and firm resource budget *T* have little impact on the whole innovation network. No matter which of the parameters *γ*, *b*, *T* we modify, out of the four strategies, the total profit of the random strategy always performs the worst, the average strategy second worst, the proportional strategy the second best and the greedy allocation strategy the best. This suggests that greedy strategy is able to bring the biggest income for the entire network.

**Fig 11:**
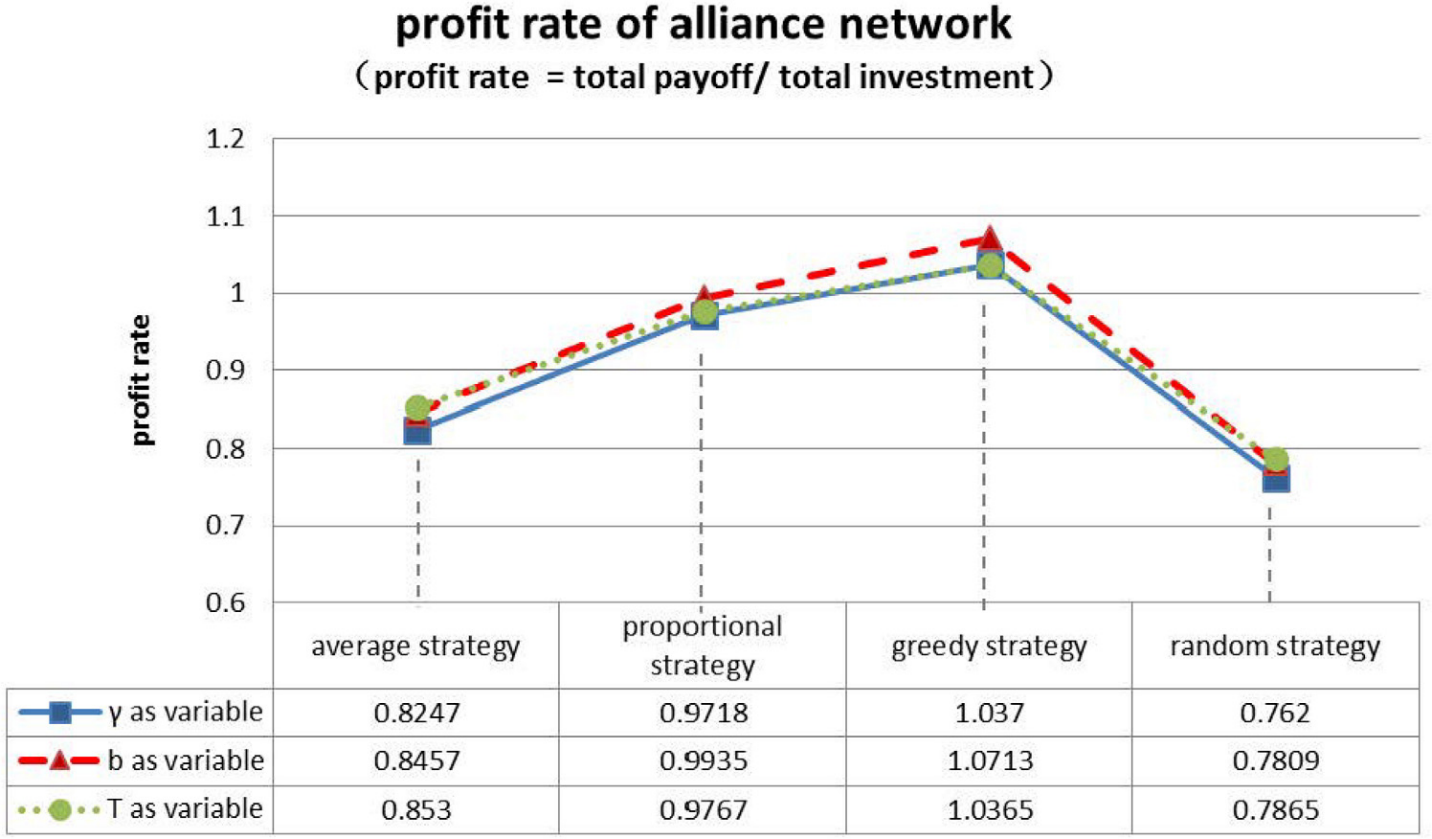
Profit rate contrast under four allocation strategies.

Thereafter, we compare the profit rate of the three groups of the initial game for each of the four strategies. The results are shown in Fig 11.

As we can see from Fig 11, changing the return ratio *γ*, complementary coefficient *b* or firm resource budget *T* again has little impact on the whole innovation network. No matter which of the parameters *γ*, *b*, *T* we modify, out of the four strategies, the total profit of the random strategy always performs the worst, the average strategy second worst, the proportional strategy the second best and the greedy allocation strategy the best. This suggests that the greedy strategy gives the highest profit rate for the alliance when the alliance’s total investment is fixed or total income is fixed.

#### Contrast and analysis of firm level

The degree of the node is the key value which captures a firm’s status in the network. The degree of the 54 firms used in the *G*_*2001*_ network is shown in Table 2. There is no firm of degree 6 within the network. In order to better relate the payoff value to the degree, the degree of 6 will not be listed in the following comparison.

**Table 2:** 54 firm node degree distribution table.

Degree of node	1	2	3	4	5	6	7
Number of firms	17	14	10	10	2	0	1

**Fig 12:**
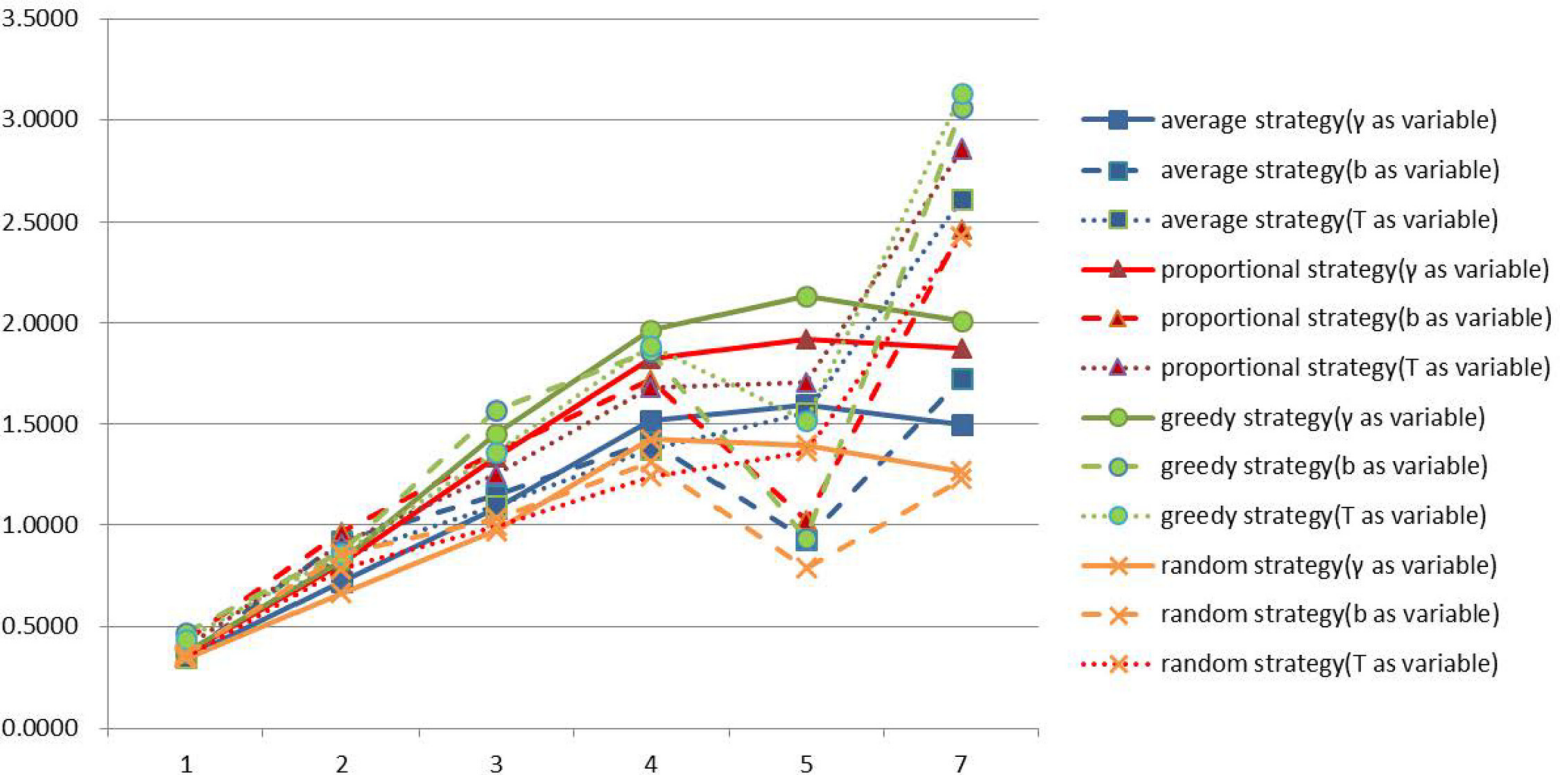
Profit expectations of different degrees of firms under four allocation strategies.

**Fig 13:**
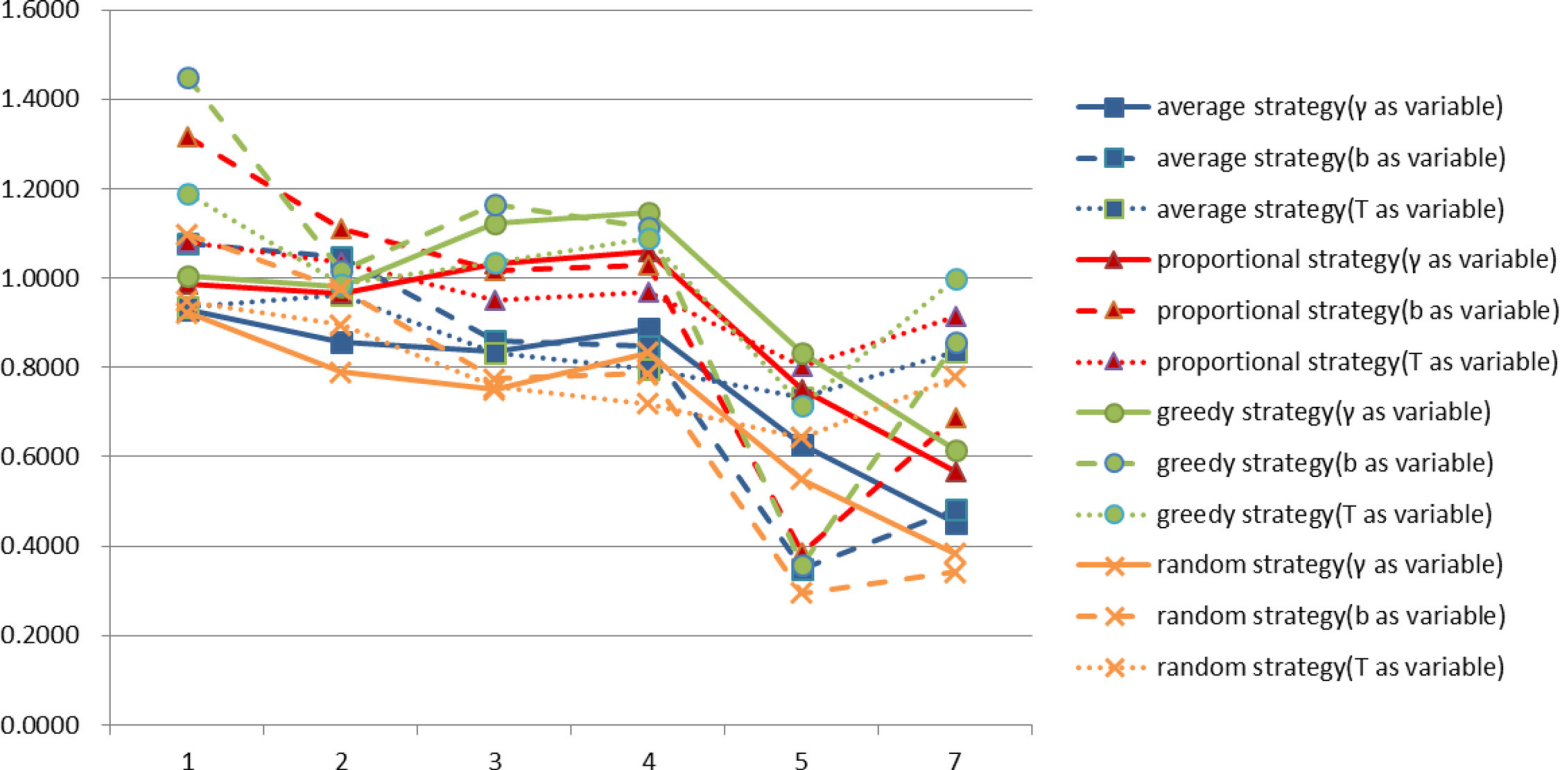
Profit rate expectations of different degree of firms under four allocation strategies.

Here, we present a comparison of the profit expectations and profit rate expectations for firms of different degrees under our four different allocation strategies. The results are shown in Figs 12 and 13 respectively.

As can be seen from Fig 12, regardless of which strategy the firms choose, firm profits and degree are positively correlated. As before, for a firm of given degree, the greedy strategy always brings the highest profit, the proportional strategy second highest, the average strategy second least and the random strategy the least profit. It can also be seen from Fig 13, that regardless of which strategy you choose, degree and profit rate are negatively related. This suggests that the higher the degree of a firm, the greater the profit of the firm but that the profit rate is not necessarily higher.

## Conclusion

Based on the above analysis, in this experimental environment in which we study innovation networks, we are able to draw the following conclusions:

(1) The greedy strategy is the most suitable resource allocation strategy in order to pursue maximum payoff for the whole network. It always brings the highest payoff for the alliance network. In second place is the performance of the proportional strategy which also performs well. This strategy is also worth considering when making allocation decisions. Although the performance of the average strategy is better than the random strategy, the average strategy is still worse than the first two strategies and is therefore not recommended.

(2) When the total investment of the alliance is a fixed value, or more extremely, the total income of the alliance is a fixed value, we need to select the appropriate strategy according to the profit rate. If the total investment of the alliance is a fixed value then selecting the greedy strategy, which has the highest profit rate, can bring a higher payoff for the alliance. If the total income of the alliance is a fixed value, then selecting the greedy strategy which has the highest profit rate can help keep the total investment for the alliance to a minimum so that the alliance has more resources available to meet other needs.

(3) The more partners a firm has the higher status the firm obtains but the more the firm has to invest into the alliance. No matter which strategy is chosen, the firms that have higher status can always obtain a higher payoff.

(4) No matter which strategy is chosen, the firms with higher status will always obtain a lower profit rate. This is because the more partnerships a firm has, the higher the costs of the investing firm, for example to maintain the partnership. Therefore, the profit rate is not high. However, for business, achieving the maximum profit rate is perhaps not necessary with a higher payoff instead being more important.

From the point of view of practical significance, the average strategy is not as good as the proportional strategy or the greedy strategy. This is because the latter two have a higher tendency to pursue the maximization of benefit than does the average strategy. The greedy strategy always seeks to allocate to the project with the highest return ratio and reduces the investment accordingly in order to obtain the highest profit rate. The proportional strategy retains the same allocation relationships as the unconstrained Nash equilibrium but it allocates less to high profit rate partners than does the greedy strategy. Thus the payoff of the proportional strategy is lower than the greedy strategy.

For possible future work, it should be noted that although the experimental environment is based on a real innovation network, some of the parameters are randomly generated. So the conclusions presented in this paper may not be applicable for all cases. In addition, the partnership game between firms still has a lot of additional factors that need to be considered and this model only gives certain experimental conclusions in a handful theoretical extreme situations to offer theoretical reasons for firms’ allocation decisions. Additionally, we believe our model could be extended to study the way in which liquidity shocks to alliance firms affect the network as a whole and whether a liquidity shock to one or more members of the alliance puts the existence of the entire alliance at risk.

## Supporting Information

S1 File Classic partnership game. (DOCX)

S2 File G2001. (CSV)

S3 File Data Description. (DOCX)
