# Supplementary material for: An Extended N-Player Network Game and Simulation of Four Investment Strategies on a Complex Innovation Network
Source: PLoS One. 2016 Jan 8;11(1):e0145407. doi: 10.1371/journal.pone.0145407 (PMC4706442; doi:10.1371/journal.pone.0145407)
Supplement: S1 File — (DOCX) [file pone.0145407.s001.docx]

# Classic partnership game model

In this supplementary file, we present a classic business partnership game and calculate the Nash equilibrium. Assume the two partners are two firms, $F_{1}$and $F_{2}$, which are cooperating on a mutual project. Assume that for this project to be successful both firms need to invest, but having done so they divide any profits equally – a kind of win-win relationship. Let the two firms have resource reserves $T_{1}$and $T_{2}$, denote both firms’ investment $S_{1}$ and $S_{2}$, and let project payoff be denoted $I$ (for income) such that

$I=4 \left( S_{1}+S_{2}+bS_{1}S_{2} \right)$ (1)

Let parameter $b,$ known as the complementary coefficient, be non-random and common knowledge among the firms. Further, let the complementary coefficient be restricted to values between 0 and $\frac{1}{4}$, that is let $b\in\left[ 0,\frac{1}{4} \right]$. From Equation (1), we see that the total payoff received by the two firms depends on both firms’ strategies $S_{1}$ and $S_{2}$ and the synergy, or cooperative effect, given by $bS_{1}S_{2}$ that is generated by the two firms working together. In reality, firms have different competitive advantages and are skilled at different projects. This is why mutual investment and cooperation may lead alliances to generate additional income, $bS_{1}S_{2}$, as compared to the income they could have generated individually. Assuming income $I$ is split equally between the two firms and that investment cost is quadratic in the level of investment, the payoff expression $P_{1}$ and $P_{2}$ corresponding to firm $F_{1}$ and firm $F_{2}$ is given by the system of equations in (2).

$\left\{ \begin{aligned} P_{1}=\frac{1}{2}*4\left( S_{1}+S_{2}+bS_{1}S_{2} \right)-S_{1}^{2} \\ P_{2}=\frac{1}{2}*4\left( S_{1}+S_{2}+bS_{1}S_{2} \right)-S_{2}^{2} \end{aligned} \right.$ (2)

In non-cooperative games, firms cannot credibly commit to a non-equilibrium level of investment, even if that non-equilibrium investment were to generate a higher total income. Given the payoff function, firms need to choose their optimal strategies $\left( \hat{S_{1}},\hat{S_{2}} \right)$ in such a way that any firm’s strategy is a best response to the other firm’s strategy. The first firm, $F_{1}$, needs to find the best response strategy $\hat{S_{1}}$ based on the strategy $S_{2}$ that firm $F_{2}$ chooses. Similarly, firm $F_{2}$ needs to find the best response strategy $\hat{S_{2}}$ based on the strategy $S_{1}$ of firm $F_{1}$. In order to find the best responses for both firms, let us first compute the first-order partial derivative of $P_{i}$ with respect to $S_{i}$, giving

$P_{i}^{'}=2\left( 1+{bS}_{j} \right)-2S_{i}$ (3)

Setting the derivatives in (3) to zero, $P_{i}^{'}=0$, we find that each firm’s best-response function is given by $S_{i}=1+bS_{j}$. Let ${BR}_{i}\left( S_{j} \right)$denote the best strategy function that $F_{i}$takes when $F_{j}$adopts the strategy $S_{j}$, then the best response functions of $F_{1}$ and $F_{2}$ are:

$\left\{ \begin{aligned} {BR}_{1}\left( S_{2} \right)=\hat{S_{1}}=1+bS_{2} \\ {BR}_{2}\left( S_{1} \right)=\hat{S_{2}}=1+bS_{1} \end{aligned} \right.$ (4)

As can be seen from (4), if $S_{i}<1+bS_{j}$, firm $F_{i}$does not have the resources to achieve the maximum, theoretical payoff. On the other hand, if $S_{i}>1+bS_{j}$, firm $F_{j}$may not be able not increase its level of investment $S_{j}$and the resources that $F_{i}$ invested are wasted. This means that only if $S_{i}=1+bS_{j}$can firm $F_{i}$ achieve the maximal payoff. Let $S_{i}^{*}$denote the Nash equilibrium solution, then we can easily verify that $S_{1}^{*}=S_{2}^{*}=\frac{1}{1-b}$. In this state, no firm can benefit by deviated from its strategy given the strategy of the other firm and hence the current set of strategies $(S_{1}^{*},S_{2}^{*})$ constitute a Nash equilibrium.

From this result, we know that when the complementary coefficient $b$ diminishes, meaning that the returns to collaboration diminish, the payoffs will decline for both sides. The firm that invests more incurs a higher marginal cost but only receives half of the marginal return.
