# Supplementary material for: An Extended N-Player Network Game and Simulation of Four Investment Strategies on a Complex Innovation Network
Source: PLoS One. 2016 Jan 8;11(1):e0145407. doi: 10.1371/journal.pone.0145407 (PMC4706442; doi:10.1371/journal.pone.0145407)
Supplement: S3 File — (DOCX) [file pone.0145407.s003.docx]

# Data Description

This readme file contains information regarding the file containing the experimental data for the innovation network of an alliance of Chinese automobile manufacturing firms belonging to the *G_2001_* network.

File name: G2001.csv

Data format: matrix, comma-separated variables

Description: Data for the innovation network of an alliance of Chinese automobile manufacturing firms.

Details:

- An adjacent matrix is used to represent the *G_2001_* network.
- Each line and row is an automobile manufacturing firm which is represented by Firm $i=1,\ldots,54$ in the data.
- The value of 1 in the adjacent matrix means there is an alliance formed between these two firms. The value of 0 means there is no alliance between the two firms.
- There are totally 54 automobile manufacturing firms and 66 alliance relationships between them.
- The last row gives the Degree of each firm.

The original data was collected from SDC Platinum supplied by Thomson Reuters (<http://thomsonreuters.com/en/products-services/financial/market-data/sdc-platinum.html>). This data is widely used in research, as in e.g. [1].

We use the alliances of Chinese automobile manufacturing firms formed during 2001 to 2003 from SDC Platinum. From the collected data we build the *G_2001_* innovation network provided in the .csv file. There are totally 54 firms that entered into an alliance between each other with 66 alliance relationships between them. For details about the construction of the network please see [2].

[1] Schilling, M. and C. Phelps (2007). "Interfirm collaboration networks: The impact of large-scale network structure on firm innovation." Management Science 53(7): 1113-1126.

[2] Zhou W., Y. Zhao, Y. Li. (2010). A fast algorithm of building interfirm collaboration network. The 2nd International Conference on Computer and Automation Engineering: 442-444.)
